# Predicting risk of psychosis in primary care: a qualitative study

**DOI:** 10.3399/BJGP.2025.0222

**Published:** 2025-12-02

**Authors:** Daniela Strelchuk, Sarah Sullivan, David Kessler, Irwin Nazareth, Katrina Turner

**Affiliations:** 1 Centre for Academic Mental Health, Population Health Sciences, Bristol Medical School, University of Bristol, Bristol, UK; 2 Department of Primary Care and Population Health, University College London, London, UK

**Keywords:** primary care, psychosis, risk, electronic health records

## Abstract

**Background:**

P Risk is a new tool that aims to help GPs identify people who are at risk of developing psychosis. It uses electronic health record data on non-psychotic symptoms, medications, and sociodemographic factors.

**Aim:**

To explore clinicians’ and patients’ views of the acceptability and usefulness of using P Risk in primary care for identifying people at risk of developing psychosis.

**Design & setting:**

Qualitative study using semi-structured interviews conducted with GPs, early intervention (EI) team clinicians, and patients between May and December 2023.

**Method:**

Participants were recruited from Bristol and London, and three topic guides were developed to ensure consistency across interviews. Interviews were transcribed verbatim and analysed thematically.

**Results:**

A total of 10 GPs, six EI clinicians, and 13 patients were interviewed. Most clinicians and patients welcomed the development of P Risk as a tool for improving the identification of people at risk of developing psychosis; however, some clinicians raised concerns about the quality of clinician coding in primary care medical records, availability of effective treatments, limited capacity of EI teams to work with people at risk, increased workload for GPs, and the negative impact on patients from being told about their risk of developing psychosis. For patients, identifying people at risk only made sense if treatment for them would be available. Interviewees said that clinicians should explain to patients what psychosis is, what it means to be at risk, which factors drive the risk, and how to address those factors.

**Conclusion:**

Although most clinicians and patients welcomed the development of P Risk, there needs to be a clear pathway for assessing patients and offering treatment to those who are identified as being at risk of developing psychosis.

## How this fits in

Identifying people at risk of developing psychosis in primary care is difficult. The P Risk algorithm uses electronic health record data on non-psychotic symptoms, medications, and sociodemographic factors to inform GPs of a patient’s risk of developing psychosis. Study participants felt that telling someone that they may be at risk of psychosis should be done by a GP whom the patient knows and trusts. Patient communication should focus on what psychosis is, the modifiable factors that drive the risk, and how to address them. A clear pathway from assessing patients to offering treatment for those identified as being at risk should be prioritised.

## Introduction

‘Psychosis’ is a term that refers to a group of severe mental health illnesses characterised by episodes of loss of contact with reality. The outcomes of psychosis can be poor; only one in seven individuals make a full recovery^1^ and, in the UK, only 5%–15% of people with schizophrenia are employed.^2^

Offering early treatment to people at risk of psychosis can decrease their risk of transitioning.^3^ Guidelines published by the National Institute for Health and Care Excellence (NICE) recommend that people at risk of psychosis are referred to early intervention (EI) for psychosis teams, or other specialist mental health services, and are offered cognitive behavioural therapy for psychosis (CBTp) with or without family intervention.^4^ EI teams were originally commissioned to offer specialised support to people experiencing their first episode of psychosis but, more recently, approximately half of the EI teams in England have also offered treatment (mostly CBTp) to people at risk of psychosis.^5^

GPs are usually the first point of contact for patients with mental health problems, and they play a key role in referring patients to secondary care. However, identifying people at risk in primary care is difficult. Possible reasons include:

- the early symptoms of psychosis are non-specific;^6^
- most GPs do not develop diagnostic skills for identifying these patients as, individually, they see few cases per year;^7^ and
- patients do not always have continuity of care with the same GP, so subtle changes in their mental state are sometimes missed.^8^

Sullivan *et al* ^9,10^ developed an algorithm, called P Risk, that aims to help GPs identify people at risk of psychosis over the next 5 years; to do this, P Risk uses electronic health record data regarding:

- non-psychotic symptoms and signs (consultations for suicidal behaviour, depression and/or anxiety, and substance misuse; history of consultations for suicidal behaviour; smoking history; prescribed medications for depression, anxiety, post-traumatic stress disorder, and/or obsessive compulsive disorder; and total number of consultations); and
- sociodemographic factors (age, sex, ethnicity, and level of social deprivation).

P Risk has good discriminative accuracy (Harrell’s *C* statistic = 0.78, meaning it can correctly discriminate between those who will, and will not, develop psychosis in 78% of cases).^9^ However, for P Risk to be used in practice, it needs to be acceptable and relevant to practitioners and patients. This study aimed to explore clinicians’ and patients’ views of the acceptability and usefulness of using P Risk in primary care for identifying people at risk of developing psychosis.

## Method

### Sampling and recruitment

It was decided that interviews should be conducted with:

- GPs — as they play a crucial role in referring patients to secondary care services;
- EI clinicians — as, according to NICE guidelines, patients at risk should be assessed by, and offered treatment in, EI teams;^4^
- patients — as P Risk uses patients’ health record data; and
- carers.

#### GP recruitment

All GP practices in Bristol and London were informed about the study via local Clinical Research Networks (CRNs). Each GP practice was asked to help recruit one or two GPs for the qualitative interviews. Eight Bristol practices originally expressed an interest, of which five signed a collaboration agreement. The other three practices either stopped replying or informed the researchers that they were no longer able to support the study. Four London practices expressed interest and signed the collaboration agreement.

#### EI recruitment

EI clinicians from four mental health trusts (one in Bristol and three in London) were informed about the study via their local CRNs. EI clinicians from three trusts expressed an interest in being interviewed.

#### Patient recruitment

Patients were recruited via the same GP practices that were involved in GP recruitment. To be eligible, patients had to be aged ≥18 years and consulted with their GP for non-psychotic mental health problems in the previous 6 months. Eligible patients were invited by their practice to take part in the study via a text message, sent to their mobile telephones, that included a link to the study webpage. Prior to sending out the text message, the list of eligible patients was reviewed by a local GP. Patients who were interested were asked to complete a form that indicated this, and return it to a researcher. The researcher then contacted patients who were interested to answer questions and to arrange a convenient date/time for the interview.

#### Carer recruitment

It was not possible for the researchers to identify any carers as patients either reported that they did not have one, or said they would ask their carer for permission to pass on their contact details to the study team but did not get back in contact.

### Data collection

Topic guides were used to ensure consistency across interviews. Two topic clinician guides (one GP and one EI clinician) and one patient guide were developed in parallel to ensure key areas were included in each. They were based on the aims of the research and developed through discussion with team members.

The clinician guide included questions about the current identification and management of patients at risk, advantages and disadvantages of using P Risk, patient communication, and treatment. The patient topic guide included questions about patients’ views of GPs using P Risk, and patient communication and treatment (see Supplementary Information S1).

Verbal consent to take part in the study and to audio-record the interviews was obtained from interviewees immediately prior to interview. Interviews were conducted via video-conference by a researcher experienced in mixed-method psychosis research; video-conferencing allowed the interviewer to present the interviewees with a prototype of P Risk.

**Figure 1.**
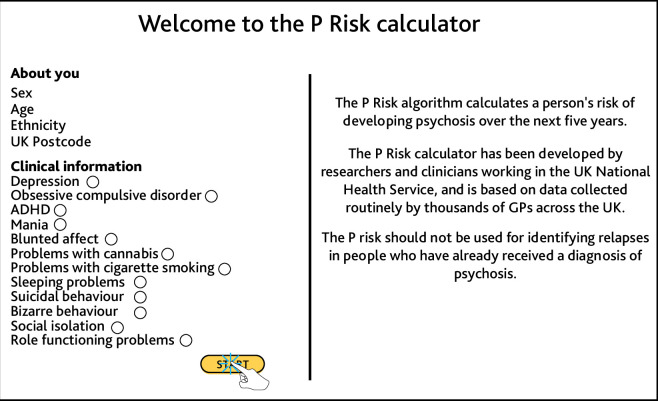
An example of the P Risk prototype, as shown to interviewees.

**Figure 2.**
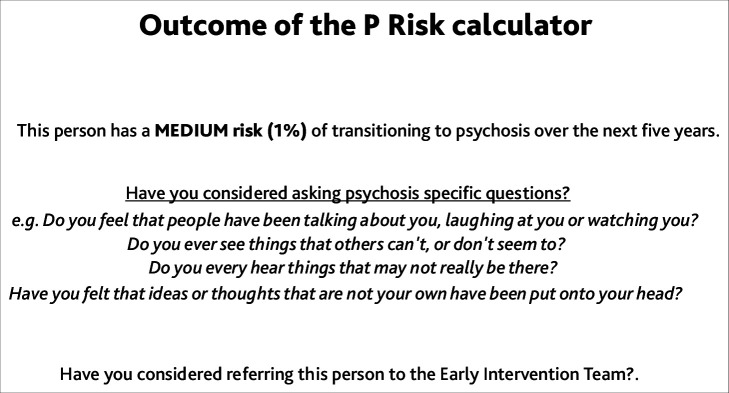
An example of the P Risk outcome, as shown to interviewees.

At the start of the interview, the researcher shared their screen with the interviewee, and showed them the P Risk prototype. The prototype illustrated the predictors used by the algorithm to calculate someone’s risk of psychosis (Figure 1). The researcher explained that the algorithm would run on GPs’ computers and automatically calculate risk when GPs entered a code for a non-psychotic mental health problem. It was explained that the GP would then be prompted to ask additional psychosis-relevant questions to patients who were identified as being at risk (Figure 2).

### Data analysis

All interviews were transcribed verbatim (except for one patient interview that was poorly recorded and for which the interviewer took notes) and analysed thematically.^11^ Analysis entailed the interviewer and another researcher independently reading and manually coding a sample of transcripts according to codes they had developed inductively. They then met to compare their coding, and create one coding framework for each set of interviews. These coding frameworks were then independently applied to another sample of transcripts, and new codes were added as needed. The same researchers then met again to discuss their coding, which resulted in further codes being added or existing codes being clarified.

When the coding frameworks were finalised, all transcripts were uploaded to NVivo (version 12) and coded electronically. Data under specific codes were then retrieved and summarised in tables to enable researchers to look across, and within, the interviews, and to highlight common themes and deviant cases. Each dataset was analysed independently before findings were compared across datasets. Findings were also discussed with other researchers and primary care clinicians in the team.

### Public and patient involvement (PPI)

The research question originated from a series of psychosis service user forum events, during which service users had described problems they had experienced getting their GP to recognise warning symptoms of psychosis. Prior to the funding application for the study presented here, the researchers held two virtual PPI events with 13 attendees from across the UK, who had lived experience of either psychosis or caring for someone with psychosis. PPI input directly informed the development of the study. After receiving funding for the study, a new PPI group (which comprised six people) further reviewed the patient-facing study documentation.

## Results

In total, the researchers interviewed 10 GPs, six EI clinicians, and 13 patients. Practitioners were interviewed between May and November 2023, and patients between August and December 2023. The interviews’ mean duration was 23 minutes for GPs, 27 minutes for EI clinicians, and 25 minutes for patients.

### Participant characteristics

Participants' characteristics are outlined in Table 1. Four (40%) GPs, four (67%) EI clinicians, and 10 (77%) patients were from Bristol. Seven (70%) GPs, four (67%) EI clinicians, and 10 (77%) patients were female. The mean age of participants was 42.4 years (standard deviation [SD] 7.9 years) for GPs, 49.5 years (SD 12.1 years) for EI clinicians, and 39.7 years (age range 20–69 years, SD 15.3 years) for patients.

**Table 1.** Interviewees’ demographic characteristics

Interviewees	Total, *N*	Practice location		Mean age, years (SD)	Female, *n* (%)
		Bristol, *n* (%)	London, *n* (%)		
GPs	10	4 (40)	6 (60)	42.4 (7.9)	7 (70)
EI clinicians	6	4 (67)	2 (33)	49.5 (12.1)	4 (67)
Patients	13	10 (77)	3 (23)	39.7 (15.3)	10 (77)

EI = early intervention. SD = standard deviation.

Of the GPs interviewed, one (10%) had an intercalated degree in psychology, and two (20%) said they saw a high number of patients with mental health problems. The mean length of time working as a GP was 11.1 years (SD 6.3 years). Of the EI clinicians, two (33%) were consultant psychiatrists, two (33%) were specialty registrars in psychiatry, one (17%) was a senior practitioner, and one (17%) was a mental health nurse (data not shown).

Of the patients interviewed, three (23%) were from a minoritised ethnic background (data not shown).

### Themes

The main themes identified were:

- advantages and disadvantages of using P Risk in primary care; and
- patient communication.

Quotations that illustrate these themes and give further insight into participants’ views have been tagged to denote the applicable group (GP, EI, and P [patient]) and research site (B [Bristol] and L [London]) of the participant. The accompanying number is the participant identifier.

### GPs’ views

#### Advantages and disadvantages of using P Risk in primary care

Most GPs said that P Risk would be a valuable tool as it would raise their awareness of patients at risk, especially in those with subtle presentations or those consulting for common mental health illnesses:

*‘It could make you think about psychosis … in patients you may not have thought about before. So sometimes in less-severe depression … with some occasional cannabis use in someone who’s quite chatty … you might not think to screen for psychosis.’* (GPL2)

A couple of GPs also said that P Risk would help them to quantify an instinct, which would then be helpful in initiating a discussion about psychosis with the patient.

Some GPs also mentioned that people at risk could be offered treatment that would target their risk factors (for example, cannabis use) and, thereby, prevent the transition to psychosis:

*‘If there is somebody who’s isolated and has a problem with cannabis, maybe we could involve the* [city] *drugs project and try and work with them more intensively to come off the cannabis … So you’re trying to deal with the risk factors and have prevention in place rather than that person then just develops a psychotic illness and you have to go down that route.’* (GPB4)

When GPs were asked whether they would trust the P Risk score, some said that they would need to know which factors had driven the individual risk, and emphasised that P Risk should not replace clinical judgement.

Some raised concerns about the quality of coding or mentioned that they had never coded for some of the predictors used by P Risk:

*‘Social isolation, not sure I would code that very often. Bizarre behaviour, I’m not sure I’ve ever coded that … Role functioning problems I didn’t even realise was a code ... Problems with cannabis … I don’t tend to code these type of things ‘cos of my concerns about them.’* (GPL1)

Some GPs also said that P Risk might prompt them to screen for psychosis in patients who did not need to be screened, and mentioned that there may not be sufficient resources to manage the additional work:

*‘My only thing would always be time … it might be the indirect time of are we asking the … psychosis questions to people who didn’t need it asked … It might be a minute per patient but a minute per patient for ten thousand patients a year might be quite a time constraint.’* (GPL2)

Several GPs also raised concerns that were related to the availability of effective treatment for people at risk:

*‘Are there any evidence-based interventions that have been shown to reduce the risk … if we can’t do anything about it then is it worth screening people for* [it]*?’* (GPL4)

Concerns about EI teams’ capacity to offer treatment and patients’ willingness to engage with it were also highlighted.

#### Patient communication

GPs were asked when, and how, patients should be told that they were at risk of developing psychosis. Their views varied: although some said patients should be told in the original consultation (*‘you don’t want to make it a massive thing that you need to prepare them for’* [GPB3]), others said that this should be communicated to patients in a follow-up consultation, especially if they were already experiencing depression or anxiety. However, one GP said that it would not be helpful to tell people they were at risk:

*‘Absolutely not … that thing about understanding yourself as a ticking time bomb, if you’re already feeling anxious … telling someone their risk of developing psychotic phenomena, it’s a bit like telling everybody in their 50s or 60s they’re at risk of developing cancer. Do I really need to think about that very much? Not sure I do.’* (GPL1)

The same GP suggested that, instead, patients should be told how to improve their wellbeing (for example, getting good sleep and abstaining from drug use).

In terms of communication strategies, most GPs said that people should, first, be told what psychosis is and signposted to written information. There should also be a focus on the modifiable factors that drove the risk, and how patients could decrease their own risk. Given the sensitivity of the topic, some GPs said that this conversation should be carried out by a GP whom the patient knows and trusts.

### EI clinicians’ views

#### Advantages and disadvantages of using P Risk in primary care

From EI clinicians’ perspectives, P Risk would be especially helpful in improving the identification of people with insidious signs of psychosis:

*‘The blatantly obvious stuff gets in quite easily, but I think sometimes the more subtle stuff gets missed and we lose an opportunity to support people early on.’* (EIB3)

EI clinicians also said that P Risk could make the risk of psychosis more real to people, and help GPs explain to patients how to decrease their risk:

*‘They* [GPs] *could … tell them* [patients] “*Look, your cannabis use puts your risk at 13% and if we take cannabis use out of the picture your risk is down to 6%”.’* (EIB4)

Some EI clinicians mentioned that, although not all EI services were commissioned to work with people at risk, developing a tool that would improve patient identification should not depend on the current commissioning situation, as this might change. Furthermore, some said that P Risk might improve the referral rates to EI teams, which could then help them build an argument for commissioning their services to work with these patients:

*‘If there is a substantive amount of ARMS* [at risk mental state] *type of patients … it might be that … we would become commissioned for this specific ARMS patient group … it would be very helpful … because some of those patients are our future patients anyway.’* (EIB2)

However, some EI clinicians said that, as the predictors of P Risk were quite common, it might flag up a high number of people and, potentially, overwhelm the EI services:

*‘My worry when I looked at it was … so many people … are going to flag up … sleep is a really big indicator for psychosis but it also goes wrong in almost every other mental health condition … I would worry that potentially if it* [P Risk] *got really widely used we could be getting a really vast number of people coming through for assessment.’* (EIL3)

Some EI clinicians also raised concerns about the quality of coding in primary care medical records, and potential discrepancies between GPs’ and secondary care clinicians’ use of terms describing mental health conditions (for example, ‘mania’):

*‘I think mania as a term isn’t always used by non-psychiatrists in the way that we use it … in primary care referrals I’ve seen the term mania sometimes being used when actually the severity of the problem is not sufficient to meet the criteria for mania … I think some of those terms are a bit ambiguous and I would have slight worries that GPs might tick or not tick them in a different way to how another rater would.’* (EIL4)

The reliability of P Risk could also be negatively impacted by patients inaccurately reporting their drug use, or P Risk not differentiating between those with a small/infrequent versus high/daily use of drugs.

One EI clinician further said that P Risk did not account for the severity of past episodes of illness, people’s insight, adherence with treatment, and the protective factors against psychosis.

#### Patient communication

Most clinicians said that patients should be told about their risk before being referred to secondary care. The exact timing would depend on the GP’s relationship with the patient, and whether the patient was help seeking.

Most EI clinicians stressed the importance of not frightening people when discussing their risk, but explaining to them how to minimise the risk and where to seek help:

*‘It’s important that it is conveyed in that positive way where people can say “Look, we’ve looked at something that’s a moveable feast here, that if you do something about these factors … maybe you can reduce that”.’* (EIB1)

It should be further explained that P Risk assesses someone’s probability of developing psychosis, and some people identified as being at risk might never develop psychosis. As one EI clinician stated:

*‘It’s trying to put it within that context of that this is about probability, this is about something that might happen and not something that will definitely happen.’* (EIB1)

### Patients’ views

#### Advantages and disadvantages of using P Risk in primary care

Overall, patients welcomed the development of P Risk as it would prompt GPs to screen for psychosis as, in their view, GPs might have limited expertise in identifying people at risk:

*‘I think it sounds really good … as someone who’s struggled with mental health issues most of my life … GPs are … general practitioners and they’re not like experts in the field … it’s a bit of reassurance that there’s like a little prompt for them to think about those kind of things.’* (PB4)

A couple of patients also mentioned that P Risk could motivate patients to address some of the risk factors for psychosis. However, like practitioners, patients linked the value of P Risk to treatment availability, arguing that its value depended on whether the patient could access treatment:

*‘It’s great that GPs would be able to identify … early signs and then be able to refer them to a service … but only provided that that service is there and actually fully funded and available for that person.’* (PB3)

Nevertheless, one patient said that, even in the context of limited treatment availability, it would still be worth developing a tool that would improve patient identification, as *‘*y*ou’ve got to start somewhere’.* (PB1)

When patients were asked about their concerns about using P Risk, some mentioned the reliability of the tool, the length of time it had been tested, the possibility of overdiagnosing people, and it not taking symptom severity into consideration:

*‘I’m pretty sure I have depression but … I’m relatively high functioning so I don’t think I would have a massive risk compared to one of my other friends who has relatively severe depression, they are going to probably be at higher risk than me regardless of whether they fit the other criteria or not.’* (PB7)

Patients also said that P Risk should be used by a GP who knows the patient and is able to assess symptoms in their context.

Furthermore, some patients were concerned about how GPs would initiate a discussion about psychosis with a patient who consulted for a different problem, and mentioned that GPs should explain to the patient why they were being asked psychosis-specific questions:

*‘If I was asked some of those* [psychosis-related] *questions and I felt like … look I’ve come in with low mood, I’m depressed and they started asking me “Are you seeing things that maybe other people aren’t seeing?”, I think I would maybe think “Do they think I’m crazy?”.’*(PL2)

#### Patient communication

Patients were asked how helpful it would be for patients to be told they were at risk. Most patients said that this would depend on the individual, and whether the risk factors were modifiable:

*‘It’s helpful if there’s things you can do to mitigate the risk. If there isn’t, then potentially you’re just kind of scaring a patient.’* (PB3)

Patients further explained that, although telling someone they were at risk would create worry, if treatment was available and the risk factors were modifiable, then they would like to know about it:

*‘I think I would find that quite terrifying, but if it meant that I could maybe have some treatment to prevent it or decrease the risk then I’d want to know about it.’* (PL2)

In terms of communication strategies, most patients said that clinicians *‘have got to be careful not to scare the patient to death’* (PB1). Patients also said that clinicians should not use the word ‘psychosis’, as it is associated with danger. Instead, it would be better if clinicians would focus on symptoms and their impact:

*‘I think psychosis is … a loaded term for a lot of people … that sounds similar to ‘psycho’ … like think of serial killers … maybe using another term … having that be phrased in a way which just talks about the symptoms … and how that would affect the person.’* (PB1)

In addition, patients said that it would be helpful if clinicians could explain to patients why they had been identified as at risk, what factors drove their risk, what treatment was available, and how effective the treatment was.

Furthermore, GPs should emphasise that being at risk does not mean the patient will definitely develop psychosis:

*‘The emphasis needs to be on the additional help that would be provided … it needs to be made clear … it’s an additional risk and it’s not a definite, you’re not definitely going to experience psychosis.’* (PB4)

Patients’ views varied with regards to when the GP should tell people they were at risk, but most stressed the importance of knowing and trusting the GP who communicated this. However, a couple of patients from a minoritised ethnic background said that it should not be the GP but the mental health specialist who should communicate this to the patient, and only after it had been confirmed that the patient was at risk:

*‘I don’t know if the GP should say that* [someone might be at risk of psychosis] *because it would be initial phases … let’s get this person assessed by a* [specialist] *team … I think that if the doctor* [GP] *said it at that stage it would be too premature.’* (PL1)

Furthermore, some patients said that people should only be told they were at risk if the risk was very high.

## Discussion

### Summary

Most GPs and EI clinicians said that P Risk would be a valuable tool for improving identification of people at risk, especially those with subtle clinical presentations. They thought that identifying patients could facilitate their being offered treatment that would target their risk factors and, potentially, prevent transition to psychosis. Overall, patients were positive about the use of P Risk, although, for them, the value of identification was directly related to treatment provision.

GPs and EI clinicians raised concerns about the quality of coding in primary care records and its potential impact on the reliability of the tool. Some GPs also raised concerns related to the availability of effective treatments, patients’ willingness to engage with treatment, and limited capacity of EI teams to work with these patients. In contrast, most EI clinicians were not particularly concerned about their capacity; on the contrary, some thought that an increase in the number of referrals might contribute towards the commissioning of EI teams to work with a high-risk patient group.

Most patients and clinicians said that telling someone they were at risk of developing psychosis was a sensitive issue that should be communicated by a GP whom the patient knows and trusts. Clinicians should tell patients what psychosis is, what it means to be at risk, what modifiable factors drive the risk, and how to address them.

### Strengths and limitations

EI clinicians had a range of clinical experience and expertise; some worked in areas where EI teams were funded to work with those at risk, others were funded to work only with patients who had already developed psychosis. It might have been helpful to also interview psychologists, as they play an important role in providing talking therapies to people at risk.

Patient interviewees were male and female, and from a range of GP practices and EI teams in Bristol and London. However, it is acknowledged that the GP practices, EI teams, and individual clinicians and patients who were interviewed were self-selecting, and may have had an interest in psychosis, which might have biased the findings.

All interviews were conducted via video-conferencing, and the richness of data collected indicates that online interviews can gather the same material as those conducted in person.^12^ The clinician and patient topic guides covered the same areas, which allowed practitioners’ and patients’ views to be compared. Internal bias in data analysis was minimised by double coding a sample of the interviews and discussing findings with other researchers and primary care clinicians.

Most patients interviewed defined themselves as White British. The researchers would like to have interviewed more people from a minoritised ethnic background, especially as, in Western countries, psychosis is more common in some minoritised ethnic groups compared with the White majority.^13^ Similarly, it would have been beneficial to have interviewed more males, as males have been found to be at higher risk of psychotic disorders compared with females.^14^

Another limitation is that it was not possible to interview any carers as had originally been planned. However, this was an important finding in itself, as it showed that some people who consulted their GP for non-psychotic mental health problems did not have any carers; it was also possible that some patients might not feel comfortable discussing mental health issues with family members.

### Comparison with existing literature

The difficulty of identifying people at risk of developing psychosis has been discussed in many research studies.^6,15–18^ A recent meta-analysis signalled the need for developing screening instruments that would take into account non-psychotic symptoms, as they are often missed by GPs; for example, it has been shown that, although GPs feel quite confident identifying the positive symptoms of psychosis (for example, delusions and hallucinations), they may miss the non-specific symptoms, such as low mood or decreased social functioning.^19^

Consistent with the findings presented here, other studies have also shown that people who consulted for common mental health symptoms might not always be screened for psychosis;^15,18,20^ this indicates why P Risk could be beneficial in terms of identifying those less-obvious cases. Similar to other studies that highlighted the complexity of coding in primary care,^21^ clinicians in this study raised concerns about the quality of coding, and its potential impact on the reliability of P Risk. It is reassuring that P Risk was developed and validated on large datasets of UK primary care data (using the Clinical Practice Research Datalink), and has been found to have good psychometric properties.^9,10^

As signalled by most participants in the study presented here, identifying people at risk of developing psychosis has to be accompanied by the provision of effective treatments. Although CBTp is an effective treatment for people at risk^3,22,23^ and is recommended by current NICE guidelines,^4^ not all people at risk have access to it — as an example, in England, only ~50% of EI teams are funded to work with these patients.^5^ The authors acknowledge this is a complex situation, and believe that, although identifying people at risk is important, there needs to be a clear pathway for assessment and treatment, especially in those EI teams that are not funded to work with patients at risk. It is therefore important to further explore other services that may offer help to the high-risk group, such as talking therapy services, drug and alcohol services, or charity organisations.

Most patients in the study presented here emphasised that telling someone they are at risk of developing psychosis is alarming and must be handled sensitively. Several studies have discussed how societal conceptions about psychosis can lead to self-stigma, fear, and negative coping behaviours that can delay the disclosure of symptoms and acceptance of treatments.^24–26^ It has also been suggested that telling people about their psychosis risk may have implications that are positive (that is, greater self-understanding and instilling hope that there is a way forward) and negative (that is, internalisation of negative stereotypes about psychosis and fear about how they may be viewed by others).^27^ Good communication is, therefore, essential when communicating risk to patients. Although the authors are not aware of any studies that have examined the effectiveness of different ways of communicating psychosis risk, it has been suggested that asking people what it means for them to be identified as being at risk, would be important.^27^ In addition, similar to another study,^28^ the interviewees of the study presented here mentioned that risk for psychosis should only be communicated by a clinician whom the patient knows and trusts.

This study’s findings are in line with the results of a recent study, which showed that patient communication should be personalised.^29^ As P Risk provides clinicians with information about which factors drive an individual’s risk score, those clinicians can then personalise information and discuss with the patient their personal risk factors and how to address them. Interestingly, a review that aimed to examine the efficacy of personalised risk estimates in changing health-related behaviours (for example, smoking and diet) suggested that the impact of communicating risk information could be much higher if clinicians addressed people’s self-efficacy (that is, perceived capacity to implement certain changes) and response efficacy (that is, the belief that a certain behaviour would reduce a certain threat).^30^

### Implications for research and practice

Most interviewees welcomed the development of P Risk as it could help GPs to identify early symptoms of psychosis that might otherwise go unnoticed. However, it is crucial that GPs are offered guidance regarding the pathway for assessing and offering treatment to those identified. Future research should examine methods for communicating risk of psychosis to patients (ideally by comparing novel communication strategies with treatment as usual), explore strategies for dealing with the modifiable risk factors, and examine the best management approach, which may be different for people at medium versus high risk. Future research should then further examine the feasibility of implementing P Risk in real-life situations. If P Risk is shown to be valid and acceptable for use in such situations, clinical services will need to adapt their criteria accordingly to ensure that those people identified as being at risk of developing psychosis are offered adequate support and treatment.
